# Effects of Oral Probiotics on *Streptococcus mutans* in Children: A Systematic Review and Meta-Analysis

**DOI:** 10.3390/dj14020087

**Published:** 2026-02-03

**Authors:** Andrea Caiza-Rennella, Andrea Ordoñez-Balladares, Rosangela Caicedo-Quiroz, Indira Gómez-Capote, Zuilen Jiménez-Quintana

**Affiliations:** 1 Carrera de Odontología, Universidad Bolivariana del Ecuador, Duran 092406, Ecuador; adcaizar@ube.edu.ec (A.C.-R.); rcaicedoq@ube.edu.ec (R.C.-Q.); 2 Facultad de Estomatología, Universidad de Ciencias Médicas de la Habana, Habana 10100, Cuba; indira@infomed.sld.cu (I.G.-C.); zuilen.jimenez@infomed.sld.cu (Z.J.-Q.)

**Keywords:** oral probiotics, *Lactobacillus reuteri*, *Lactobacillus rhamnosus*, *Streptococcus mutans*, early childhood caries, oral microbiota, pediatric dentistry

## Abstract

**Background**: Early childhood caries is closely associated with oral dysbiosis and the proliferation of *Streptococcus mutans*. Oral probiotics, particularly *Lactobacillus reuteri* and *Lactobacillus rhamnosus*, have been proposed as ecological modulators capable of reducing cariogenic microorganisms. **Objective**: To evaluate the efficacy of orally administered *L. reuteri* and *L. rhamnosus* in reducing salivary *S. mutans* levels in children aged 6 months to 12 years through a systematic review and meta-analysis. **Methods**: This review followed the PRISMA 2020 guidelines and was prospectively registered in PROSPERO (CRD420251086304). Searches were conducted in MEDLINE/PubMed, CENTRAL, Embase, Scopus and LILACS without language or date restrictions. Randomized controlled trials administering the target probiotic strains for ≥30 days were included. Risk of bias was assessed using RoB 2, and certainty of evidence using GRADE. Random-effects meta-analyses were performed for continuous and dichotomous outcomes. **Results**: Six randomized controlled trials were included (N = 1362). Only two trials reported continuous outcomes in comparable log_10_ CFU/mL format and could therefore be pooled for the continuous meta-analysis. This analysis showed a significant reduction in salivary *S. mutans* levels (MD = −0.65 log_10_ CFU/mL; 95% CI: −0.97 to −0.34; *p* < 0.0001; I^2^ = 19%), although the pooled estimate was largely driven by one study and should be interpreted cautiously. Four trials contributed to the dichotomous meta-analysis, which showed a non-significant trend toward risk reduction (OR = 0.73; 95% CI: 0.51–1.06; *p* = 0.10; I^2^ = 35%). Short-term interventions using high oral-retention formulations demonstrated the most consistent microbiological effects. **Conclusions**: Oral probiotics may significantly reduce salivary *S. mutans* in the short-term, especially when delivered through slow-dissolving formulations. However, their effects vary according to strain, vehicle, and intervention duration. Larger, standardized, and longer-term clinical trials are needed to determine the sustainability and clinical relevance of these effects.

## 1. Introduction

Dental caries is one of the most prevalent chronic diseases in childhood and represents a major global public health concern, being associated with pain, recurrent infections, impaired quality of life, and increased healthcare costs [1,2,3,4]. Despite extensive preventive efforts, childhood caries remains highly prevalent worldwide and continues to impose a substantial burden on healthcare systems [5,6].

Traditionally considered an infectious and transmissible disease, contemporary evidence supports an ecological model in which caries is understood as a biofilm-mediated dysbiosis driven by environmental and behavioral factors [5,7,8]. This ecological concept, originally formalized by Marsh and later expanded by Zaura and Twetman, recognizes that oral health depends on maintaining a balanced relationship between the host and the oral microbiota, and that frequent exposure to fermentable carbohydrates can shift the ecosystem toward acidogenic and aciduric communities capable of promoting dental demineralization [9,10,11].

Among the microorganisms associated with cariogenic dysbiosis, *Streptococcus mutans* has been consistently linked to the initiation and progression of early carious lesions [9,12,13,14] and is widely used as a surrogate microbiological marker in clinical research. Nevertheless, dental caries is a polymicrobial disease in which other acidogenic and aciduric species such as *Streptococcus sobrinus*, *Lactobacillus* spp., *Actinomyces* spp., and anaerobic genera including *Bifidobacterium*, *Prevotella*, and *Veillonella* also contribute to lesion development [7,11,14]. Therefore, changes in *S. mutans* levels should be interpreted as indicators of ecological modulation rather than direct clinical outcomes.

Conventional preventive strategies, including mechanical plaque control, topical fluorides, and antimicrobial agents, can reduce pathogenic bacterial load but may not restore long-term microbial homeostasis [8,15,16]. In this context, oral probiotics have emerged as a potential adjunctive strategy for modulating the oral ecosystem and reducing cariogenic risk [17,18]. Probiotic strains such as *Lactobacillus reuteri* and *Lactobacillus rhamnosus* have demonstrated the ability to inhibit cariogenic bacteria through antimicrobial metabolite production, competitive adhesion, coaggregation with pathogens, and immunomodulatory mechanisms [19,20,21,22,23,24,25]. However, clinical trials evaluating oral probiotics in children have reported heterogeneous findings, which appear to depend on strain specificity, delivery vehicle, dosage, and duration of administration [26,27,28,29,30,31].

Previous systematic reviews and meta-analyses have pooled diverse probiotic strains and formulations without consistently distinguishing strain-dependent effects, vehicle-related oral retention, or short- versus long-term interventions [32,33,34,35,36]. Consequently, uncertainty remains regarding which probiotic approaches provide reproducible microbiological benefits in pediatric populations. The present systematic review and meta-analysis aims to address these unresolved aspects by synthesizing evidence on oral probiotic interventions in children, with particular attention to strain characteristics, delivery vehicles, and intervention duration, and by quantitatively evaluating their effects on salivary *Streptococcus mutans* levels as indicators of oral ecological modulation.

## 2. Materials and Methods

### 2.1. Protocol and Registration

This systematic review and meta-analysis were conducted in accordance with the Preferred Reporting Items for Systematic Reviews and Meta-Analyses (PRISMA 2020) guidelines [37]. The completed PRISMA 2020 checklist is provided as Supplementary File S1. A protocol was prospectively registered in the International Prospective Register of Systematic Reviews (PROSPERO; registration ID: CRD420251086304) to ensure methodological transparency and reduce the risk of bias.

### 2.2. PICO Question

The research question was formulated according to the PICO model:

- Population (P): Children aged 6 months to 12 years.
- Intervention (I): Oral administration of probiotics containing exclusively *Lactobacillus reuteri* and/or *Lactobacillus rhamnosus* for ≥30 days.
- Comparator (C): Placebo or identical products without viable microorganisms.
- Outcome (O): Quantitative levels of *Streptococcus mutans* in saliva or dental plaque, expressed as CFU/mL, log_10_ CFU/mL, or the proportion of participants with high bacterial counts (≥10^5^ CFU/mL). Plaque-based outcomes were planned to be analyzed separately if sufficient studies were available.

The overarching question was: *Does oral administration of L. reuteri or L. rhamnosus reduce Streptococcus mutans levels in children compared with placebo?*

### 2.3. Database Search Strategy and Screening

A comprehensive electronic search was conducted on 24 August 2025 across CENTRAL, Embase (Ovid and Embase.com), LILACS, MEDLINE, PubMed, and Scopus, without restrictions on language or publication date. Google Scholar was used only for supplementary screening of potentially missed records and was not considered a primary source database. The core search strategy included the following terms and their combinations using Boolean operators (AND/OR): *Lactobacillus reuteri*, *Lacticaseibacillus rhamnosus*, oral probiotics, *Streptococcus mutans*, dental caries, oral microbiota, child, preschool, pediatric, infant. All retrieved references were imported into Rayyan (Qatar Computing Research Institute), which facilitated blinded independent screening by two reviewers and resolution of conflicts. The electronic search and initial study identification were independently conducted by two reviewers (A.C.R. and A.O.B.).

Additionally, supplementary manual searches were performed to identify potentially relevant trials published in regional journals not indexed in major international databases.

### 2.4. Eligibility Criteria

*Inclusion Criteria*

Studies were included if they met all of the following criteria:

- Randomized controlled clinical trials (parallel-group or cluster designs).
- Participants aged 6 months to 12 years, healthy or at risk for caries.
- Interventions consisted of oral administration of *Lactobacillus reuteri* and/or *Lactobacillus rhamnosus* in any formulation (lozenges, drops, sachets, dairy products, or fortified foods).
- A comparator group receiving placebo or an identical vehicle without viable microorganisms.
- Minimum probiotic intervention duration of 30 days.
- Studies reporting quantitative *Streptococcus mutans* outcomes in saliva or dental plaque.
- No restriction on publication year or language.

*Exclusion Criteria*

Studies were excluded if they met any of the following criteria:

- Non-randomized, quasi-experimental, or observational study designs.
- In vitro or animal studies.
- Probiotics not including the *L. reuteri* or *L. rhamnosus*.
- Probiotic intervention duration shorter than 30 days.
- Absence of quantitative *Streptococcus mutans* outcome data.
- Presence of co-interventions (e.g., antimicrobial mouthrinses or systemic antibiotics) that prevented isolation of the probiotic effect.

### 2.5. Study Selection Process and Data Extraction

Two independent reviewers (A.C.R. and A.O.B.) systematically performed the literature search, study selection, and screening process. In the first phase, titles and abstracts of all retrieved records were screened for relevance. Subsequently, the full texts of potentially eligible studies were assessed to confirm final inclusion. Any disagreements were resolved by consensus, and when necessary, a third reviewer (I.G.C.), blinded to the primary hypothesis, was consulted to ensure objectivity and minimize selection bias.

Data extracted from each included RCT comprised: first author, year of publication, country, participants’ age range, study design, total sample size and group allocation, probiotic strain administered (*L. rhamnosus* or *L. reuteri*), route and duration of administration, comparator type, microbiological method used for quantification of *Streptococcus mutans* (CFU/mL in saliva or dental plaque), and caries-related clinical outcomes (incidence or increment). In addition, main findings reported by the authors and the reviewers’ interpretation regarding consistency and clinical relevance were documented.

### 2.6. Risk of Bias Assessment

The risk of bias in the included randomized controlled trials was independently assessed by two reviewers (A.C.R. and A.O.B.) using the Cochrane Risk of Bias 2 (RoB 2) tool. The following domains were evaluated: randomization process, deviations from intended interventions, missing outcome data, measurement of outcomes, and selection of reported results. Discrepancies were resolved through discussion or consultation with a third reviewer (I.G.C.). Each study received a judgment for each domain (low risk, some concerns, or high risk) and an overall risk-of-bias assessment.

### 2.7. Statistical Analysis

Quantitative synthesis was performed using random-effects meta-analysis models to account for expected clinical heterogeneity among included studies. Pooled estimates were calculated using the DerSimonian–Laird method. For continuous outcomes, salivary *Streptococcus mutans* concentrations expressed as log_10_ CFU/mL were analyzed using mean differences (MDs) with 95% confidence intervals (CIs). For dichotomous outcomes, defined as the proportion of participants with high salivary *Streptococcus mutans* loads (≥10^5^ CFU/mL), odds ratios (ORs) with corresponding 95% confidence intervals (CIs) were calculated.

Statistical heterogeneity was assessed using the I^2^ statistic and Cochran’s Q test and interpreted according to Cochrane Handbook recommendations. Visual inspection of funnel plots was considered when appropriate. All statistical procedures followed Cochrane methodological guidelines for systematic reviews of interventions.

Given the expected clinical diversity in probiotic strains, dosages, and delivery vehicles, a random-effects model was intentionally applied to estimate an overall pooled effect while accounting for inter-study heterogeneity. The purpose of combining different probiotic formulations was to obtain a global estimate of probiotic efficacy rather than strain-specific efficacy, which could not be analyzed separately due to limited study numbers.

### 2.8. Certainty of Evidence

The certainty of the evidence for each outcome was assessed using the GRADE approach, considering the following domains: risk of bias, inconsistency, indirectness, imprecision, and publication bias. Evidence profiles and summary of findings tables were constructed accordingly.

## 3. Results

### 3.1. Study Selection

The electronic search identified a total of 1320 records from the selected databases: PubMed (*n* = 219), Embase (*n* = 283), Scopus (*n* = 342), Web of Science (*n* = 294), Cochrane Library (*n* = 166), and LILACS (*n* = 16).

After removal of 718 duplicates and 42 records excluded for other reasons, 560 records were screened based on titles and abstracts. Of these, 521 were excluded for not addressing the research question. Thirty-nine full-text articles were assessed for eligibility, and 33 were excluded for the following reasons:

- Insufficient duration of probiotic exposure (*n* = 10);
- Use of probiotic strains not meeting the inclusion criteria (*n* = 3);
- Study population outside the predefined age range (*n* = 3);
- Non-randomized or non-experimental study design (*n* = 5); and
- Absence of quantitative microbiological outcomes for *Streptococcus mutans* (*n* = 12).

**Figure 1:**
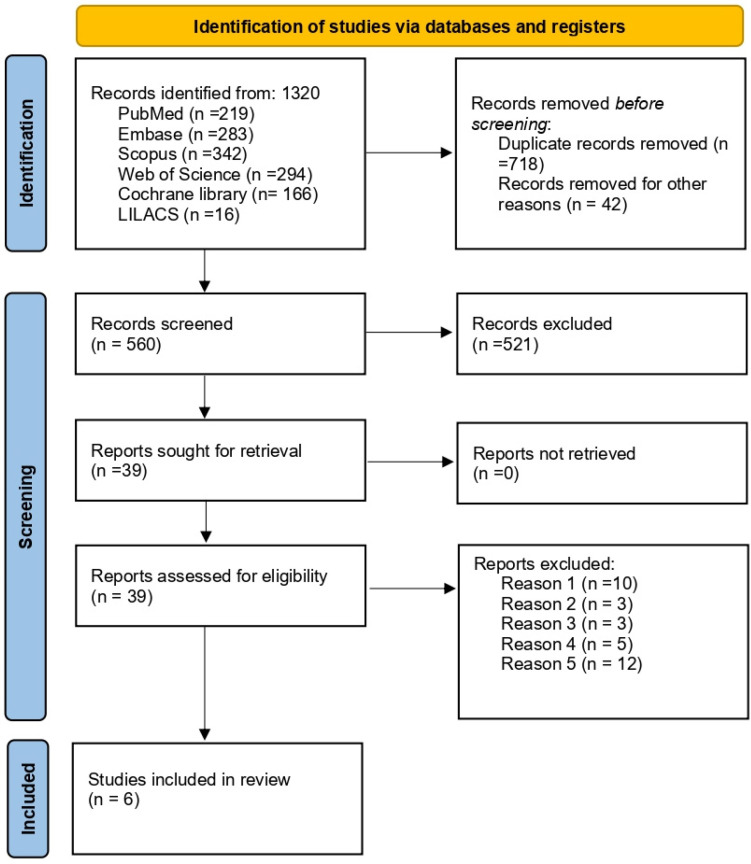
PRISMA flowchart.

Ultimately, six randomized controlled trials were included in both the qualitative and quantitative syntheses. The complete study selection process is illustrated in the PRISMA flow diagram Figure 1.

### 3.2. Characteristics of Included Studies

Six randomized controlled trials evaluated the effectiveness of different probiotic strains of *Lactobacillus rhamnosus* and *Lactobacillus reuteri* on salivary *Streptococcus mutans* levels and caries-related outcomes in pediatric populations (N = 1362) [26,27,28,29,30,31]. The studies were conducted between 2001 and 2025 across diverse geographic settings and included children aged 1 to 12 years.

All trials comprised an experimental group receiving oral probiotic supplementation (*n* = 699) and a control group receiving an identical product without viable microorganisms (*n* = 663) [26,27,28,29,30,31]. The probiotic interventions varied in strain, formulation, dosage, and duration. Three studies reported statistically significant reductions in salivary *S. mutans* following probiotic administration [26,27,28], whereas the remaining trials did not demonstrate significant microbiological effects [29,30,31]. Detailed methodological characteristics and outcomes of the included trials are summarized in Table 1.

Specifically, three studies reported significant reductions in *S. mutans* concentrations following administration of *L. rhamnosus* SD11 or *L. reuteri* DSM17938 combined with ATCC PTA5289 [26,27,28]. In contrast, three trials employing long-term administration of probiotic-supplemented milk or juice containing *L. rhamnosus* GG or LB21 did not demonstrate statistically significant changes in *S. mutans* levels, despite observing favorable clinical trends in caries outcomes in some cases [29,30,31].

### 3.3. Results of Risk of Bias Assessment

**Figure 2:**
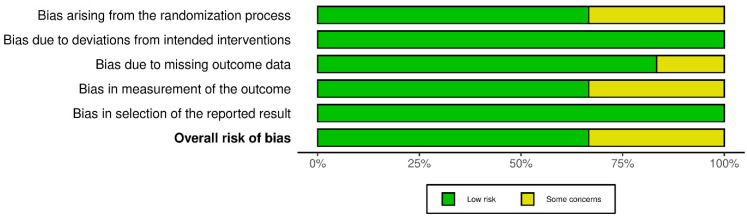
Risk-of-bias summary by domain (RoB 2).

**Figure 3:**
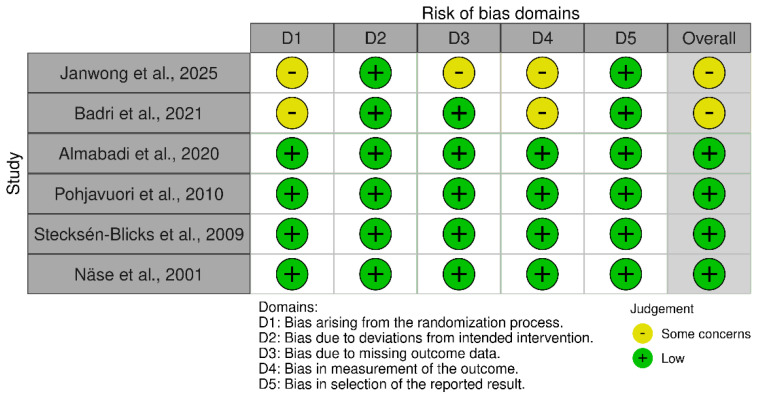
Traffic light plot of risk of bias by study and domain (RoB 2), including Janwong et al., 2025 [26]; Badri et al., 2021 [27]; Almabadi et al., 2020 [28]; Pohjavuori et al., 2010 [29]; Stecksén-Blicks et al., 2009 [30]; and Näse et al., 2001 [31].

Overall, four of the six included randomized controlled trials were judged to have a low overall risk of bias, whereas two studies presented some concerns, primarily related to the randomization process and blinding of outcome assessors. Across all trials, the domains addressing deviations from intended interventions and selective reporting of results demonstrated a consistently low risk of bias. The risk-of-bias assessment was conducted using the Cochrane Risk of Bias 2 (RoB 2) tool [26,27,28,29,30,31,37]. The domains most frequently rated as “some concerns” were the randomization process and blinding of outcome assessment, whereas deviations from intended interventions and selective reporting were consistently judged at low risk. A detailed summary of the risk-of-bias judgments by domain and by study is presented in Figure 2 and Figure 3.

### 3.4. Individual Study Results

Table 2 presents the individual results of the included studies. For continuous outcomes, defined as salivary *Streptococcus mutans* levels expressed as CFU/mL or log_10_ CFU/mL, the studies by Janwong et al. [26] and Badri et al. [27] demonstrated statistically significant reductions in favor of the probiotic groups.

For dichotomous outcomes, defined as the proportion of children with high salivary *S. mutans* levels, only the study by Almabadi et al. [28] showed a statistically significant effect favoring probiotic administration, whereas the remaining trials did not reach statistical significance [29,30,31].

### 3.5. Results of Quantitative Synthesis

**Figure 4:**
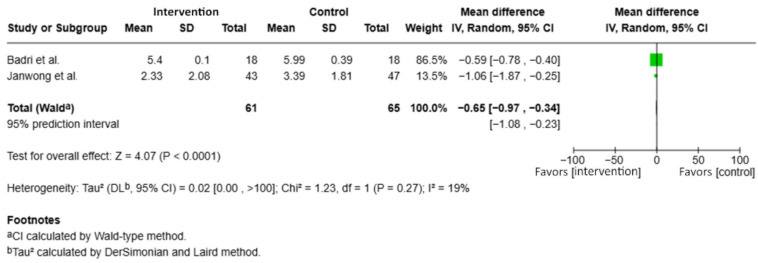
Forest plot of mean salivary *Streptococcus mutans* concentrations (log_10_ CFU/mL) comparing probiotic and control groups, including Badri et al., 2021 [27] and Janwong et al., 2025 [26].

The meta-analysis of continuous outcomes (Figure 4) included two studies [26,27] reporting means salivary *Streptococcus mutans* concentrations expressed as log_10_ CFU/mL following probiotic intervention. Only one included study reported *S. mutans* outcomes in dental plaque. As no other trials provided comparable plaque-based data, a separate meta-analysis for plaque outcomes was not feasible, and pooled analyses were therefore limited to salivary outcomes. However, only two studies contributed data to this pooled analysis, which limits the robustness of the estimated mean difference and warrants cautious interpretation of the magnitude of effect.

The pooled analysis showed a statistically significant mean difference (MD) of −0.65 (95% CI: −0.97 to −0.34; *p* < 0.0001), indicating a significant reduction in *S. mutans* levels in the probiotic group compared with the control group.

No substantial heterogeneity was observed between the included studies (I^2^ = 19%; *p* = 0.27), supporting the consistency of the results. The study by Badri et al. [27] contributed the greatest weight (86.5%) to the pooled estimate due to its smaller standard deviation, thereby exerting a dominant influence on the overall effect size. The pooled estimate was largely driven by one study with lower variance, emphasizing that the statistical significance should be interpreted cautiously. Taken together, these findings indicate that probiotic administration is associated with a significant reduction in salivary *S. mutans* bacterial load.

**Figure 5:**
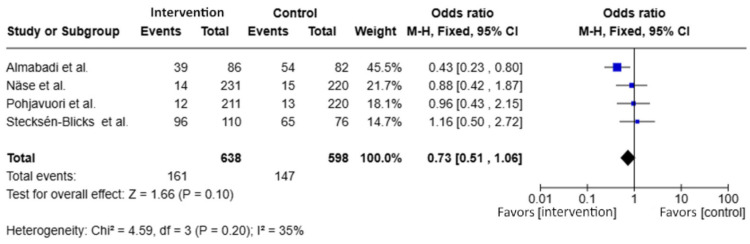
Forest plot of the proportion of children with high salivary *Streptococcus mutans* counts (≥10^5^ CFU/mL), including Almabadi et al., 2020 [28]; Näse et al., 2001 [31]; Pohjavuori et al., 2010 [29]; and Stecksén-Blicks et al., 2009 [30]. The blue squares represent the point estimates of individual studies, with the square size proportional to the study weight.

For dichotomous outcomes (Figure 5), four studies [28,29,30,31] reporting the proportion of participants with high *S. mutans* counts (≥10^5^ CFU/mL) were included in the meta-analysis. The pooled effect estimate showed an odds ratio (OR) of 0.73 (95% CI: 0.51 to 1.06; *p* = 0.10), representing a non-significant trend toward risk reduction in the probiotic group compared with the control group.

Heterogeneity was low to moderate (I^2^ = 35%; *p* = 0.20), indicating acceptable consistency across studies. Among the included trials, Almabadi et al. [28] demonstrated a statistically significant individual effect (OR = 0.43; 95% CI: 0.23–0.80), whereas the remaining studies showed non-significant estimates in the same direction of effect.

### 3.6. Sources of Heterogeneity

The observed heterogeneity (I^2^ = 19–35%) may be explained by differences in probiotic strain and mode of administration. Studies evaluating *Lactobacillus reuteri* showed more consistent effects, whereas those using *Lactobacillus rhamnosus* administered in milk reported variable results depending on follow-up duration.

Short-term interventions (30 days to 3 months) were associated with statistically significant reductions in *Streptococcus mutans* levels, whereas longer interventions (7 to 21 months) did not demonstrate significant differences between groups. This pattern suggests that probiotic efficacy may be greater with short-term exposure, possibly due to a transient colonization effect and reduced persistence or microbial compensation during prolonged treatments.

Additionally, trials using *L. reuteri* delivered through slow-dissolving formulations consistently showed short-term reductions in *S. mutans*, whereas long-term interventions using *L. rhamnosus* in milk-based vehicles did not demonstrate sustained microbiological effects, suggesting strain- and follow-up-dependent differences in efficacy.

### 3.7. Sensitivity Analysis

A sensitivity analysis was not performed for continuous outcomes (mean salivary *Streptococcus mutans* concentrations expressed as log_10_ CFU/mL) due to the limited number of available studies (*n* = 2). Both studies presented some concerns related to randomization and blinding of outcome assessors; therefore, exclusion of either study would have precluded pooled estimation.

Consequently, it was not possible to assess the robustness of the effect with respect to exclusion of studies at risk of bias. Nevertheless, the low observed heterogeneity (I^2^ = 19%) suggests stability of the pooled results.

### 3.8. Publication Bias

Publication bias was not assessed due to the limited number of available studies (*n* < 10) for each outcome (continuous and dichotomous).

### 3.9. Results of Certainty of Evidence

The certainty of the evidence was evaluated using the Grading of Recommendations Assessment, Development and Evaluation (GRADE) approach. Among the analyzed outcomes, salivary *Streptococcus mutans* demonstrated a moderate level of certainty, as both randomized controlled trials presented some concerns related to allocation concealment and/or blinding of outcome assessors, while showing consistency across results and adequate statistical precision (Table 3). The domains most frequently rated as “some concerns” in RoB 2 (randomization process and blinding of outcome assessment) were consistent with the single-level downgrading applied in the GRADE certainty assessment.

The outcome assessing the proportion of children with high *S. mutans* loads (≥10^5^ CFU/mL) demonstrated a low level of certainty, primarily due to risk of bias in some studies and imprecision of the confidence interval, which crossed the line of no effect. Overall, the evidence suggests that oral probiotics may reduce salivary *S. mutans* load in the short-term; however, the overall certainty of the evidence remains limited to confirm a sustained long-term clinical effect (Table 3).

## 4. Discussion

The evidence synthesized in this systematic review and meta-analysis indicates that oral probiotics may significantly modulate the pediatric oral ecosystem, with measurable effects on *Streptococcus mutans* (SM) levels and caries progression. While only six trials were included in the quantitative synthesis, additional external trials are cited in this discussion to provide contextual support and are not part of the pooled estimates. However, the consistency of these effects varies according to probiotic strain, delivery vehicle, and duration of administration. These findings are consistent with previous clinical studies and meta-analyses by Meng et al. [32], Shi et al. [33], Panchbhai et al. [34], Talib et al. [35], and Zhang et al. [36], which reported overall reductions in SM levels and moderate clinical benefits, albeit with substantial heterogeneity. This variability reflects differences in probiotic strain, dosage, delivery vehicle, and duration of intervention.

Among the trials included in the present review, the most consistent effects were observed in interventions employing probiotic strains with direct antimicrobial activity. Janwong et al. [26] demonstrated that *Lactobacillus rhamnosus* SD11 significantly reduced salivary SM counts and the incidence of new carious lesions. Similar findings were reported by Almabadi et al. [28] and Badri et al. [27], in which *Lactobacillus reuteri* administered in slow-dissolving tablets produced significant reductions in SM levels over short intervention periods. A dose–response relationship could not be established, as included trials used heterogeneous probiotic concentrations and dosing regimens. These results are concordant with external clinical trials conducted by Wattanarat et al. [38], Villavicencio et al. [39], Patil et al. [40], Kamble et al. [41], Ebrahim et al. [42], Starck et al. [43], and Salim et al. [44], which also demonstrated reductions in SM counts in children or adolescents using strains such as L. paracasei SD1, Streptococcus salivarius M18, or combinations of *L. reuteri*. Although short-term reductions in *S. mutans* were observed, microbiological changes do not necessarily translate into sustained clinical caries prevention.

In contrast, studies based on probiotic-supplemented milk, such as those by Rodríguez et al. [45] and Sandoval et al. [46], reported clinical benefits—including reduced caries progression or modulation of immune peptides such as human β-defensin-3 (hβD-3)—without proportional reductions in SM levels. Similarly, classical trials by Näse et al. [31] and Stecksén-Blicks et al. [30] observed reductions in caries risk associated with probiotic milk consumption but failed to demonstrate a direct effect on SM counts. These findings suggest that liquid vehicles, due to their rapid clearance from the oral cavity, limit the contact time between probiotics and dental biofilm, thereby reducing their localized antimicrobial activity.

The trends observed in the present review are consistent with current meta-analytic evidence. Meng et al. [32] reported that *L. rhamnosus* reduces caries incidence and progression and lowers the prevalence of high salivary SM loads. Shi et al. [33] demonstrated a significant pooled effect in favor of probiotics (standardized mean difference ≈ −1.17), whereas Panchbhai et al. [34] documented consistent reductions in caries outcomes among preschool children. Talib et al. [35] reported beneficial effects on both microbial and gingival parameters in individuals aged 1–18 years. Finally, Zhang et al. [36] highlighted the ability of probiotics to interfere with biofilm formation and reduce key caries-associated factors.

These findings support an ecological modulation effect rather than a direct anti-caries effect, reinforcing that microbial shifts represent changes in oral ecosystem balance rather than definitive disease prevention. None of the included trials reported industry funding, and data extraction in this review was performed independently by two blinded reviewers.

### 4.1. Strain Specificity

Probiotic strains such as *L. rhamnosus* SD11, *L. reuteri* DSM17938, and *S. salivarius* M18 have demonstrated consistent inhibitory effects on SM [26,27,28,38,39,40,41,42,43,44]. In contrast, strains such as *L. rhamnosus* GG, used by Näse et al. [31], or LB21, employed by Stecksén-Blicks et al. [30], have shown clinical effects on caries outcomes but limited direct suppression of SM. These findings reinforce the concept that probiotic efficacy in caries prevention is highly strain dependent.

### 4.2. Delivery Vehicle

Delivery vehicles with high oral retention, such as lozenges, tablets, and fortified solid foods, provide prolonged contact with the dental biofilm and may explain the more robust effects observed in trials by Almabadi et al. [28], Badri et al. [27], and Wattanarat et al. [38]. In contrast, liquid vehicles such as milk have been associated with more modest effects on SM levels [30,31,45,46], likely due to rapid oral clearance.

### 4.3. Duration and Persistence of the Effect

Several studies, including the synthesis by Coqueiro et al. [47], indicate that reductions in SM levels tend to diminish after discontinuation of probiotic administration, suggesting that probiotic colonization of the oral cavity is transient rather than permanent. This highlights the importance of continuous or repeated administration strategies to sustain microbiological benefits.

The combined evidence supports the use of oral probiotics as adjunctive agents in the prevention of childhood caries, particularly in children at high caries risk. Trials such as those by Rodríguez et al. [45] and Sandoval et al. [46] demonstrate that clinical benefits may occur even in the absence of significant reductions in SM levels, which is consistent with the multifactorial nature of dental caries and the broader modulatory role of probiotics within the oral ecosystem.

Notably, substantial heterogeneity exists among the included studies, including differences in probiotic strains, dosages, intervention duration, microbiological assessment methods, diagnostic criteria, and control of dietary factors [3,26,27,28,30,31,32,33,34,35,36,38,39,40,41,42,43,44,45,46,47,48,49,50,51]. This heterogeneity underscores the need for longer-term, standardized, and multicenter randomized clinical trials to clarify the sustained clinical relevance of probiotic interventions in pediatric caries prevention.

### 4.4. Limitations of the Review

The methodological characteristics of the current evidence base should be interpreted within the context of an emerging research area. The modest number of trials available for meta-analysis reflects the limited but growing body of high-quality clinical studies on oral probiotics in children. Variability in strains, delivery vehicles, dosages, intervention durations, and microbiological techniques represent the real-world diversity of probiotic research rather than a weakness of the present review. Importantly, this systematic synthesis clarifies these inconsistencies and delineates clear research priorities, including the standardization of probiotic protocols and longer-term follow-up. These considerations strengthen the relevance of the present findings and provide direction for advancing pediatric probiotic research. These methodological and clinical differences highlight the need for more standardized and robust future trials.

Another limitation is that the primary outcome was microbiological, and different quantification techniques for *Streptococcus mutans* were used across studies, which may contribute to measurement variability. Moreover, microbiological changes do not necessarily translate into sustained clinical caries prevention

## 5. Conclusions

The findings of this systematic review and meta-analysis indicate that oral probiotics based on *Lactobacillus reuteri* and *Lactobacillus rhamnosus* can significantly reduce salivary *Streptococcus mutans* levels in children, particularly when administered in formulations with high oral retention and over short intervention periods. Nevertheless, the observed effects were heterogeneous and dependent on the probiotic strain, delivery vehicle, and duration of exposure.

The long-term clinical impact of probiotic use on dental caries remains inconclusive. Overall, probiotics should be considered adjunctive strategies within comprehensive caries prevention programs rather than substitutes for established conventional interventions. The results suggest that the type of probiotic formulation may influence efficacy, although further standardized trials are required.

## Figures and Tables

**Table 1:** Characteristics of Included RCTs.

Study (Year)	Country/Age	Design/*n* (Final)	Intervention (Strain, Route, Duration)	Comparator	Outcome Measure	Main Effect on *S. mutans*	Authors’ Conclusion	Reviewer’s Interpretation
Janwong et al. [26]	Thailand/2–5 years	RCT (*n* = 90)	Milk powder with *L. rhamnosus* SD11, oral, 3 months	Milk without probiotic	Salivary *S. mutans* and *Lactobacillus* (log_10_ CFU/mL); Caries incidence	↓ Significant	SD11 reduced *S. mutans* and caries incidence	Consistent with probiotic inhibition of *S. mutans* and caries
Badri et al. [27]	Saudi Arabia/8–12 years	RCT (three-arm) (*n* = 36 used for probiotic vs. control)	*L. reuteri* DSM17938 + ATCC PTA5289 lozenges, 30 days	Placebo lozenge (separate CHX arm not pooled)	Salivary *S. mutans* count (CFU/mL)	↓ Significant	*L. reuteri* lozenges significantly reduced *S. mutans*	Consistent with probiotic inhibition of *S. mutans*
Almabadi et al. [28]	Saudi Arabia/3–6 years	Double-blind RCT (*n* = 168)	*L. reuteri* lozenges, twice daily, 56 days	Placebo lozenge	Salivary *S. mutans*, streptococci, and *Lactobacillus* (log_10_ CFU/mL)	↓ Significant	*L. reuteri* lozenges effectively reduced *S. mutans*	Consistent with probiotic inhibition of *S. mutans*
Pohjavuori et al. [29]	Finland/3–6 years	RCT (*n* = 431)	Carrot-pineapple juice with *L. rhamnosus* GG, 9 months	Identical juice without probiotic	Salivary *S. mutans* (CFU/mL)	No effect/Not significant	No significant difference observed	No clear probiotic effect on *S. mutans*
Stecksén-Blicks et al. [30]	Sweden/1–5 years	Cluster-RCT (*n* = 186)	Milk supplemented with *L. rhamnosus* LB21, 21 months	Non-fortified milk	Salivary *S. mutans* (CFU/mL); Caries increment (Δdmfs index)	No effect/Not significant	No significant difference observed	No clear probiotic effect on *S. mutans* or caries
Näse et al. [31]	Finland/1–6 years	Double-blind RCT (*n* = 451)	Milk with *L. rhamnosus* GG, 7 months	Milk without probiotic	Salivary and plaque *S. mutans* (CFU/mL); Caries incidence	No effect/Not significant (for *S. mutans*)	Long-term probiotic milk reduced caries prevalence	No clear probiotic effect on *S. mutans*

**Note:** ↓ indicates a reduction in *Streptococcus mutans* levels.

**Table 2:** Individual study results.

Continuous Outcomes (Salivary *Streptococcus mutans* Levels)								
Author/Year	N (I/C)	Follow-Up Duration	Mean (I)	SD (I)	Mean (C)	SD (C)	Effect Estimate (95% CI)	Brief Interpretation
Janwong et al. [26]	43/47	3 months	2.33	2.08	3.39	1.81	MD = −1.06 (−1.87 to −0.25)	Favors probiotic
Badri et al. [27]	18/18	30 days	5.40	0.10	5.99	0.39	MD = −0.59 (−0.78 to −0.40)	Favors probiotic
**Dichotomous Outcomes (Proportion of Children with High *S. mutans* Levels)**								
**Author/Year**	**N (I/C)**	**Follow-Up Duration**	**Events (I)**	**N (I)**	**Events (C)**	**N (C)**	**Effect Estimate (95% CI)**	**Brief Interpretation**
Almabadi et al. [28]	86/82	56 days	39	86	54	82	OR = 0.43 (0.23 to 0.80)	Favors probiotic
Pohjavuori et al. [29]	211/220	7 months	12	211	13	220	OR = 0.96 (0.43 to 2.15)	Not significant
Stecksén-Blicks et al. [30]	110/76	21 months	96	110	65	76	OR = 1.16 (0.50 to 2.72)	Not significant
Näse et al. [31]	231/220	7 months	14	231	15	220	OR = 0.88 (0.42 to 1.87)	Not significant

**Table 3:** Certainty of the evidence (GRADE).

Outcome	Effect Estimate (95% CI)	No. of Participants (Studies)	Certainty (GRADE)	Justification
Salivary *Streptococcus mutans* load (log_10_ CFU/mL) at end of follow-up	MD −0.65 (95% CI: −0.97 to −0.34), favors probiotic, statistically significant (*p* < 0.05)	126 (2 RCTs)	Moderate⨁⨁⨁◯	Downgraded by one level due to risk of bias: both RCTs presented some concerns related to allocation concealment and/or unclear blinding of outcome assessors. No downgrading for inconsistency (I^2^ = 19%). PICO question appropriately aligned. No imprecision, as the 95% CI does not include the null effect. Publication bias could not be assessed (*n* < 10 studies).
Proportion of children with high *S. mutans* load (≥10^5^ CFU/mL)	OR 0.73 (95% CI: 0.51 to 1.06), trend favoring probiotic, not statistically significant (*p* ≥ 0.05)	1236 (4 RCTs)	Moderate⨁⨁⨁◯	No downgrading for risk of bias, as all four RCTs were judged to be at low risk. Downgraded by one level due to imprecision, as the 95% CI crossed the line of no effect. No downgrading for inconsistency (I^2^ = 35%, low to moderate). PICO question appropriately aligned. Publication bias could not be assessed (*n* < 10 studies).

**Note: ⊕ indicates the level of certainty according to the GRADE approach; ○ indicates a downgrade in certainty.**

## Data Availability

The original contributions presented in this study are included in the article and supplementary material. Further inquiries can be directed to the corresponding author.

## Supplementary Materials

The following supporting information can be downloaded at: https://www.mdpi.com/article/10.3390/dj14020087/s1, File S1: PRISMA 2020 Checklist.

## Abbreviations

The following abbreviations are used in this manuscript:

MD	Mean difference
OR	Odds ratio
CI	Confidence interval
RCT	Randomized controlled trial
SM	*Streptococcus mutans*
CHX	Chlorhexidine
ECC	Early childhood caries
CFU	Colony-forming units
GRADE	Grading of Recommendations Assessment, Development, and Evaluation
I^2^	Inconsistency index
PRISMA	Preferred Reporting Items for Systematic Reviews and Meta-Analyses
RoB 2	Cochrane Risk of Bias tool version
